# Proteomics Analysis of Alfalfa Response to Heat Stress

**DOI:** 10.1371/journal.pone.0082725

**Published:** 2013-12-06

**Authors:** Weimin Li, Zhenwu Wei, Zhihong Qiao, Zinian Wu, Lixiang Cheng, Yuyang Wang

**Affiliations:** 1 College of Animal Science and Technology, Yangzhou University, Yangzhou, China; 2 Institute of Grassland Research of Chinese Academy of Agricultural Sciences, Hohhot, China; 3 Research & Testing Center, Gansu Provincial Key Laboratory of Aridland Crop Science, Gansu Agricultural University, Lanzhou, China; 4 Testing Center, Yangzhou University, Yangzhou, China; Aligarh Muslim University, India

## Abstract

The proteome responses to heat stress have not been well understood. In this study, alfalfa (*Medicago sativa* L. cv. Huaiyin) seedlings were exposed to 25°C (control) and 40°C (heat stress) in growth chambers, and leaves were collected at 24, 48 and 72 h after treatment, respectively. The morphological, physiological and proteomic processes were negatively affected under heat stress. Proteins were extracted and separated by two-dimensional polyacrylamide gel electrophoresis (2-DE), and differentially expressed protein spots were identified by mass spectrometry (MS). Totally, 81 differentially expressed proteins were identified successfully by MALDI-TOF/TOF. These proteins were categorized into nine classes: including metabolism, energy, protein synthesis, protein destination/storage, transporters, intracellular traffic, cell structure, signal transduction and disease/defence. Five proteins were further analyzed for mRNA levels. The results of the proteomics analyses provide a better understanding of the molecular basis of heat-stress responses in alfalfa.

## Introduction

Temperature is one of the most crucial environmental factors determining plant growth and development. The overall global temperature is steadily increasing due to rapid increases in atmospheric greenhouse gas concentrations [1]. Higher growing season temperatures have dramatic impacts on agricultural productivity, farm incomes, and food security [2]. High temperature affected germination capacity, leading to seed cell death [3], scorching of leaves and twigs, sunburns on leaves, branches and stems, leaf senescence and abscission, shoot and root growth inhibition, fruit discoloration and damage and reduced yield [4]. Thus, the selection of heat-tolerance alfalfa varieties suited to high temperature is becoming a key breeding objective in many countries.

To understand the modulation mechanisms of heat tolerance in alfalfa, a study of the proteome in response to high temperature is essential. Proteomics offers a powerful approach to discover the proteins and pathways that are crucial for stress responsiveness and tolerance [5]. 2-DE in combination with MS allows rapid and reliable protein identification [6], [7]. In recent years, proteomic approaches have been widely used to the systematic study of the responses to a wide range of abiotic stresses, such as cold [8], [9], oxidative stress [10], drought [11], [12] and salt [13]. Most components of the heat stress response mechanism in plants also can be identified through high-throughput proteomic analysis [14], [15]. Lee at al (2007) found that heat shock proteins (Hsps) and antioxidant enzymes were up-regulated under heat stress in rice leaves [14]. Zou at al (2011) reported that in *Agrostis* a moderate heat response involves changes in proteins related to protection proteins, protein biosynthesis, protein degradation, energy and carbohydrate metabolism, and redox homeostasis [15]. Zhang et al (2013) found that major proteins such as Hsps, and energy, metabolism, redox homeostasis and signal transduction associated proteins were affected significantly by high temperature [16].

Alfalfa has a cold tolerant but heat sensitive characteristics so that yields dramatically decline in summer. Alfalfa (*Medicago sativa* L. cv. Huaiyin) is a landrace that is grown in the Jianghuai area of China, where it has a 200 year history of cultivation. It has gradually become a special local variety that is suited to a hot and humid environment. In the present study, we determined the effects of heat shock in the protein expression level. The aim of our study was to analyze leaf proteomes of up- and down-regulated proteins using different databases, in order to contribute to knowledge of molecular mechanisms underlying alfalfa tolerance/resistance to high temperatures.

## Materials and Methods

### Plant Material and Growth Conditions

Alfalfa (*Medicago sativa* L. cv. Huaiyin) seeds were scarified in concentrated anhydrous sulphuric acid and sterilized in 50% (v/v) sodium hypochloryde. Afterwards they were immersed in 70% (v/v) ethanol for 2 min, rinsed with distilled water. Sterilized seeds were grown in Murashige & Skoog solid medium within a 500 mL triangle-bottle, respectively. After 3 days at 4°C in dark conditions, triangle-bottles were transferred to the growth chamber (thermo period of 25/20°C, day/night, relative humidity of approximately 40%, and a photoperiod of 16 h light 3,000 lx alternating with 8 h darkness). Each triangle-bottle contained ∼3 seedlings. Four-week-old plants were used for heat-stress treatments. Seedlings were exposed to 40°C for 24, 48 and 72 h respectively, and seedlings grown at 25°C were used as a control. Leaf samples were harvested after treatments. All experiments were performed with three replicates, four triangle-bottles containing twelve seedlings was one replicate.

### Relative Water Contents (RWC)

For determination of RWC, 0.5 g fresh leaves were detached from each treatment and replicate, and weighed immediately to record fresh weight (FW), after which the sample was dipped in distilled water for 12 h. The leaves were blotted to wipe off excess water, weighed to record fully turgid weight (TW), and subject to oven drying at 70°C for 24 h to record the dry weight (DW). The RWC were determined by the equation: RWC = [FW-DW] / [TW-DW] × 100.

### Proline Content

To determine free proline level, 0.5 g of leaf samples from each group were homogenized in 3% (w/v) sulphosalycylic acid and then homogenate filtered through filter paper. Mixture was heated at 100°C for 1 h in water bath after addition of acid ninhydrin and glacial acetic acid. Reaction was then stopped by ice bath. The mixture was extracted with toluene and the absorbance of fraction with toluene aspired from liquid phase was read at 520 nm. Proline concentration was determined using calibration curve and expressed as μmol proline (g FW)^−1^.

### Preparation of Total Protein Extracts

Total proteins of leaves were extracted by trichloroacetic acid-acetone method. 0.5 g leaf was ground in liquid N_2_ using a mortar and pestle. The powdered tissue was placed in 50 mL tubes and then precipitated at −20°C with 10% (w/v) trichloroacetic acid in acetone containing 0.07% (w/v) 2-mercaptoethanol, after vortexing samples incubated at −20°C for 45 min. Proteins were recovered by centrifuged at 14,000 g for 15 min. The protein pellets were washed twice with a cold solution of 90% acetone containing 0.07% 2-mercaptoethanol to remove residual trichloroacetic acid, and then the pellets were washed again with 0.2% DTT instead of 2-mercaptoethanol. After air-dried and the protein was resuspended in 2-DE solubilization buffer consisting of 7 M urea, 2 M thiourea, 4% CHAPS, 1% DTT,2% Ampholytes. The concentration was quantified by the Bradford method using a commercial dye reagent (Bio-Rad) and bovine serum albumin as the standard.

### 2-DE

Proteins (600 µg per sample) were first separated by isoelectrofocusing (IEF). Proteins were separated using gel strips forming an immobilized nonlinear 3 to 10 pH gradient (IPG strip, 17 cm; Bio-Rad). Strips were rehydrated in the Protein IEF Cell (Bio-Rad), passive rehydration 4 h and active rehydration 12 h at 20°C. IEF was performed at 20°C in the Protein IEF Cell system for 1.5 h at 250 V, 1.5 h at 1,000 V, volt gradient to 9,000 V in 5 h, and 9,000 V for 90,000 volt-hours (vhr). Before the second dimension, each gel strip was incubated at room temperature for 2×15 min, in 2×5 mL equilibration solution as described by Gallardo et al (2002) [17]. Proteins were then separated in vertical polyacrylamide gels (12% (w/v) acrylamide) in a PAC 3000 Electrophoresis system (Bio-Rad). Electrophoresis was run at 12°C for 1.5 h at 70 V, then at 4°C, 200 V until the dye reached the bottom of the gel.

### Protein Staining and Analysis of 2-D Gels

Gels were stained with Coomassie Brilliant Blue G-250(CBB) (Bio-Rad, Hercules, CA) (Candiano et al 2004) [18]. Image acquisition was done using a Sharp JX-330 scanner (Amersham Biosciences) with a resolution of 300ppi and an optical density range from 0.05 to 3.05. Image analysis was done on Coomassie Blue gels with the PDQuest 8.0.1(Bio-Rad) with the following procedures: spot detection, spot measurement, background subtraction, and spot matching. Only spots that were detected on all the three replicate gels were analyzed further. To correct the variability due to staining, the spot volumes were normalized as a percentage of the total volume of all spots on the gel. The normalized percentage volumes (volume %) of protein spots from triplicate biological samples were subjected to statistical analysis using SPSS (version 20.0). Analysis of variance (ANOVA) was used to test for the effects of different heat stressed times. Means were separated by least significance difference test (*P* <0.05).

### Trypsin Digestion and Gel Extraction

Selected protein spots were excised manually from the CBB-stained gels and washed twice with milli-Q water for 15 min, followed by four washes of 25 mM NH_4_HCO_3_ in 50% acetonitrile to remove all stain from gel pieces and each wash should be 30 minutes in length. Then, the gel pieces were washed and dehydrated with 50 µL of acetonitrile until gel pieces turn opaque and dramatically shrinks in size. The gel particles were dehydrated in oven at 60°C to complete dryness and rehydrated with trypsin digestion buffer (50 mM NH_4_HCO_3_). For trypsin digestion 12.5 ng/μL of trypsin (Promega, Madison, USA) was added in a final volume of 10 µL. Tubes were incubated on ice for 45 min, then 25 mM NH_4_HCO_3_ was added and tubes were further incubated overnight at 37°C. The supernatant was removed into a clean siliconized tube and extracted three times in 50% acetonitrile contented 0.1% trifluoracetic acid. The mixture was vortexed and vacuum-dried. For MALDI-TOF/TOF, peptides were eluted into the target with 0.7 µL matrix solution, α-cyano-4-hydroxy-cinnamic acid (Sigma, St. Louis, MO, USA) in 0.1% trifluoracetic acid, 50% acetonitrile.

### Mass Spectrometry

All samples were analyzed using a 5800 MALDI TOF/TOF analyzer (Applied Biosystem, Framingham, MA, USA). Mass spectra (m/z 800–4000) were acquired in positive ion reflector mode. The 20 most intense ions were selected for subsequent MS/MS sequencing analysis in 2 kV modes. Protein identification was performed by searching MS/MS spectra and peptide mass were searched for in the NCBI protein databases using the search engine Matrix Science (http://www.matrixscience.com). The following search parameters were applied: Viridiplantae (Green Plants) chosen as taxonomy, peptide tolerance of 1.2 Da, MS/MS tolerance of 0.6 Da and one incomplete cleavage were allowed and oxidation of methionine as modification. According to the MASCOT probability analysis (*P*<0.05), only significant hits were accepted for protein identification. For the identified proteins, their sub-cellular location and functional information were extracted from software TargetP (http://www.cbs.dtu.dk/services/TargetP/) or PSORT (http://psort.hgc.jp/). In addition, the pathways and biological reactions possibly were involved were searched from Uniprot (http://www.uniprot.org/) and GenomeNet (http://www.genome.ad.jp/kegg/).

### RNA Extraction, cDNA Synthesis and PCR

Total RNA of alfalfa leaf tissue was extracted with an RNA extraction kit according to the manufacturer's protocol (TaKaRa Biotechnology, Dalian, China). Each sample was analyzed individually and processed in triplicate. RNA samples were treated with RNase-free DNase I and cDNA was synthesized from 1 µg total RNA using a cDNA synthesis kit (Takara Biotechnology, Dalian, China). The synthesized cDNA was used for PCR for the corresponding genes of identified protein spots. Reaction contained selected couples of the following gene-specific primers: Hsp17F, 5′-TGCTTGTTATAAGCGGAGAG-3′, Hsp17R, 5′-ACAGTCAACACACCATCTTG-3′; APXF, 5′-CCTTTCGGAACCATCAA-3′, APXR, 5′-CAACAACACCAGCCAACT-3′; Hsp23F, 5′-CTTCCCGTTCATTCAACACC-3′, Hsp23R, 5′-GAATCCTCTGTCTCTTTCGC-3′; Hsp90F. 5′-GCTTGTGAAAAACTACTCCC-3′, Hsp90R, 5′-GATTACGAAGCCAGATAGGTT-3′; NBPF, 5′-CTTGAGTTTATTAGCCGTGC-3′, NBPR, 5′-ACCGAGTTCTAAACACTTCC-3′; ActinF, 5′-TGGTGACGAAGCCCAATCAA-3′, ActinR, 5′-CACAATACCAGTTGTACGACCA-3′. Semi-quantitative PCR was performed in a 20 µL reaction volume. 1 µL cDNA was used as template, and all reactions were performed in triplicate. The PCR thermo-cycling was 2 min at 94°C and 30 cycles of 50 s at 94°C, 50 s at 55°C, 60 s at 72°C; the final step was 10 min at 72°C and hold at 4°C using thermocycler (Eppendorf, Germany). The housekeeping gene, Actin was used as internal control. The amplification products were detected on 0.2% agarose gels, stained with Gold wave and UV-visualized with the Gel Doc XR (Bio-rad, USA). Semi-quantification was performed by densitometry using the Quantity one (Bio-Rad) 4.62 software.

### Statistical Analysis

All experiments were repeated three times independently. Spot intensities of differential proteins in a 2D gel were calculated from three spots in three replicate gels. Physiological parameters, as well as spot intensities, were statistically analyzed using a one-way analysis of variance and Duncan's multiple range test to determine significant differences among group means. Significant differences from control values were determined at the *P*<0.05 level.

## Results

### Phenotypes and Physiological Characterization of Heat Stress in Alfalfa

**Figure 1:**
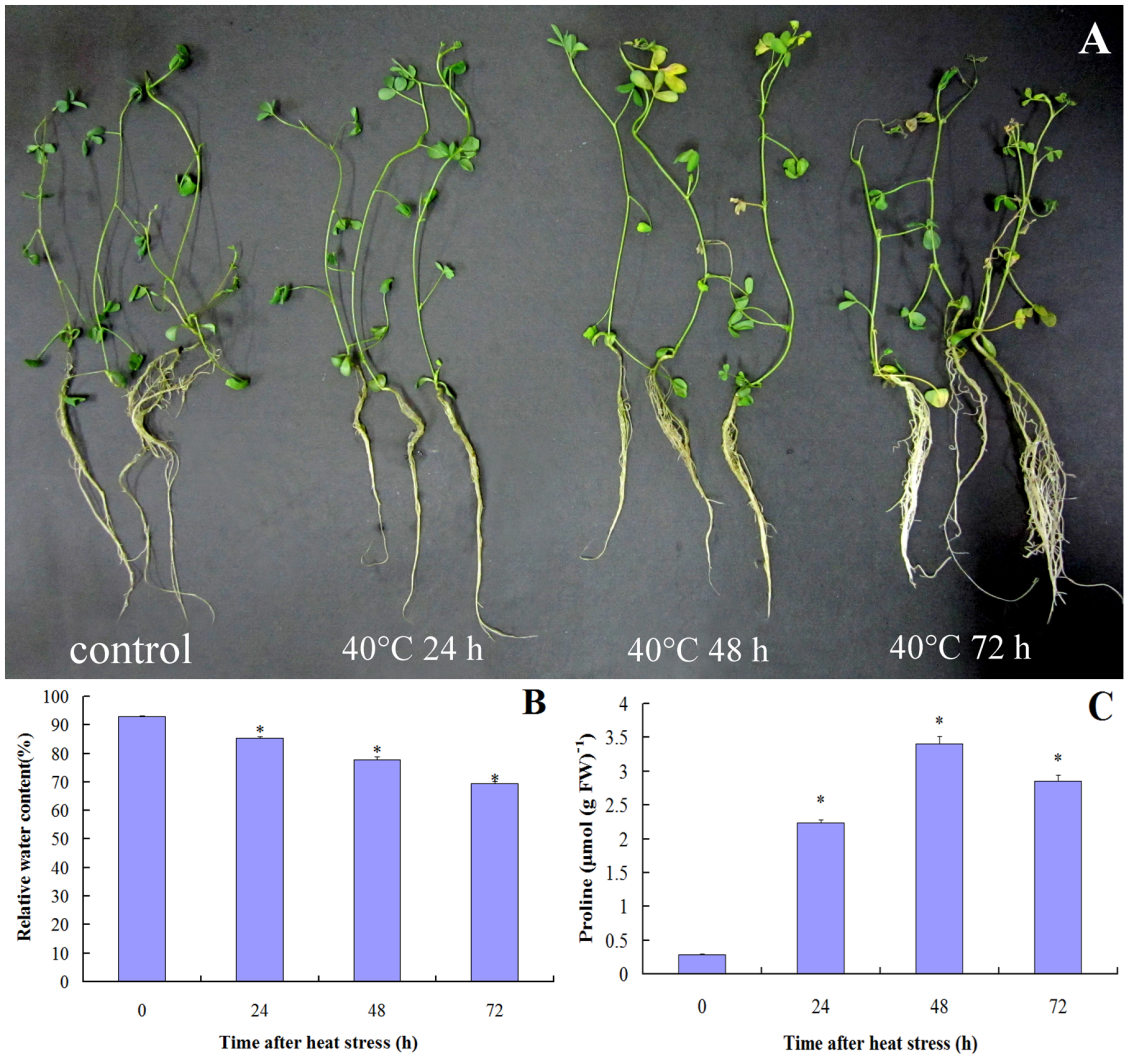
Phenotypic and physiological changes in alfalfa seedlings under control and heat stress (40°C for 24, 48 and 72 h). (A). Phenotypic changes (B). RWC (C). Proline content. Three independent biological replicates were used in each treatment. Asterisks indicate statistically significant differences at *P*<0.05. Bars represent standard errors of triplicate experiments.

Figure 1 shows the effects of heat stress on phenotypes, RWC and proline content in alfalfa. Exposure to heat stress induced phenotypic trait differences in 48 and 72 h. Leaves turning yellow were found at 48 h. Leaves and twigs were scorched with exposure time of 72 h. In contrast, no phenotypic effects were found in 24 h heat treatment. Heat stress can significantly affect the RWC, which decline along with the heat treatment time. Comparing to control, the analysis of proline content in leaves showed that the amount of proline increased after heat stress.

### 2-DE Analysis of Alfalfa Leaf Proteins under Heat Stress

**Figure 2:**
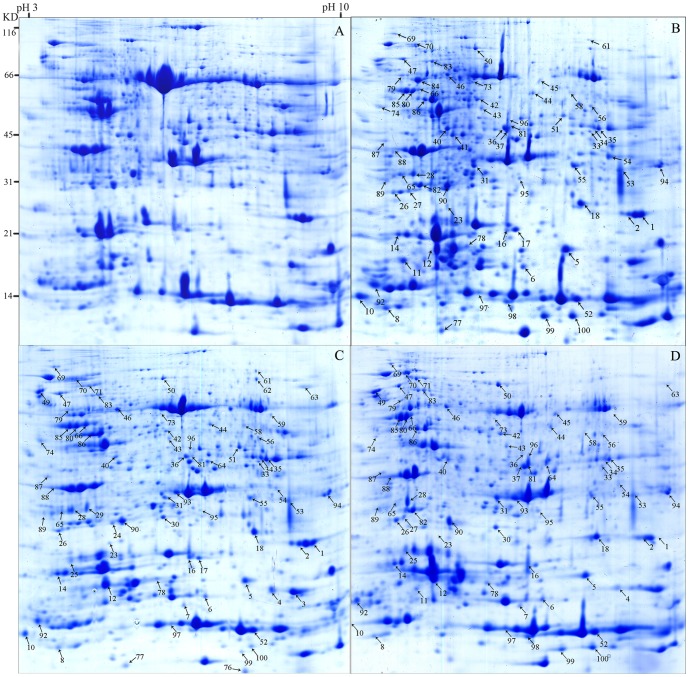
2-DE analysis of alfalfa leaf proteins extracted from alfalfa leaves grown under control(A), heat stress (40°C for 24(B), 48(C) and 72 h(D)) samples. A total of 600 µg proteins were separated by 2-DE as described in the material and methods and then visualized using CBB stain. Arrows indicate proteins that were 1.5 fold changes in respond to heat stress.

To investigate the temporal changes of alfalfa leaf proteins in response to heat stress, a time series of heat-treated samples were analyzed by 2-DE. For this purpose, total protein of 40°C treated leaves from alfalfa was extracted at following time points: 24, 48 and 72 h respectively, and 25°C treated samples were used as the control. The protein samples were separated by 2-DE (Figure 2). A quantitative analysis using PDQuest software revealed that a total of 96 protein spots were differentially identified and their intensities changed by more than 1.5-fold. Some of the identified proteins were shown either as unknown and hypothetical proteins, or without specific functions. To gain functional information about these proteins, BLASTP was used to investigate their homologies with other proteins in the database. Identified proteins showing more than 50% positive with the homologs at the amino acid level indicate that they might have a similar function [14].

**Figure 3:**
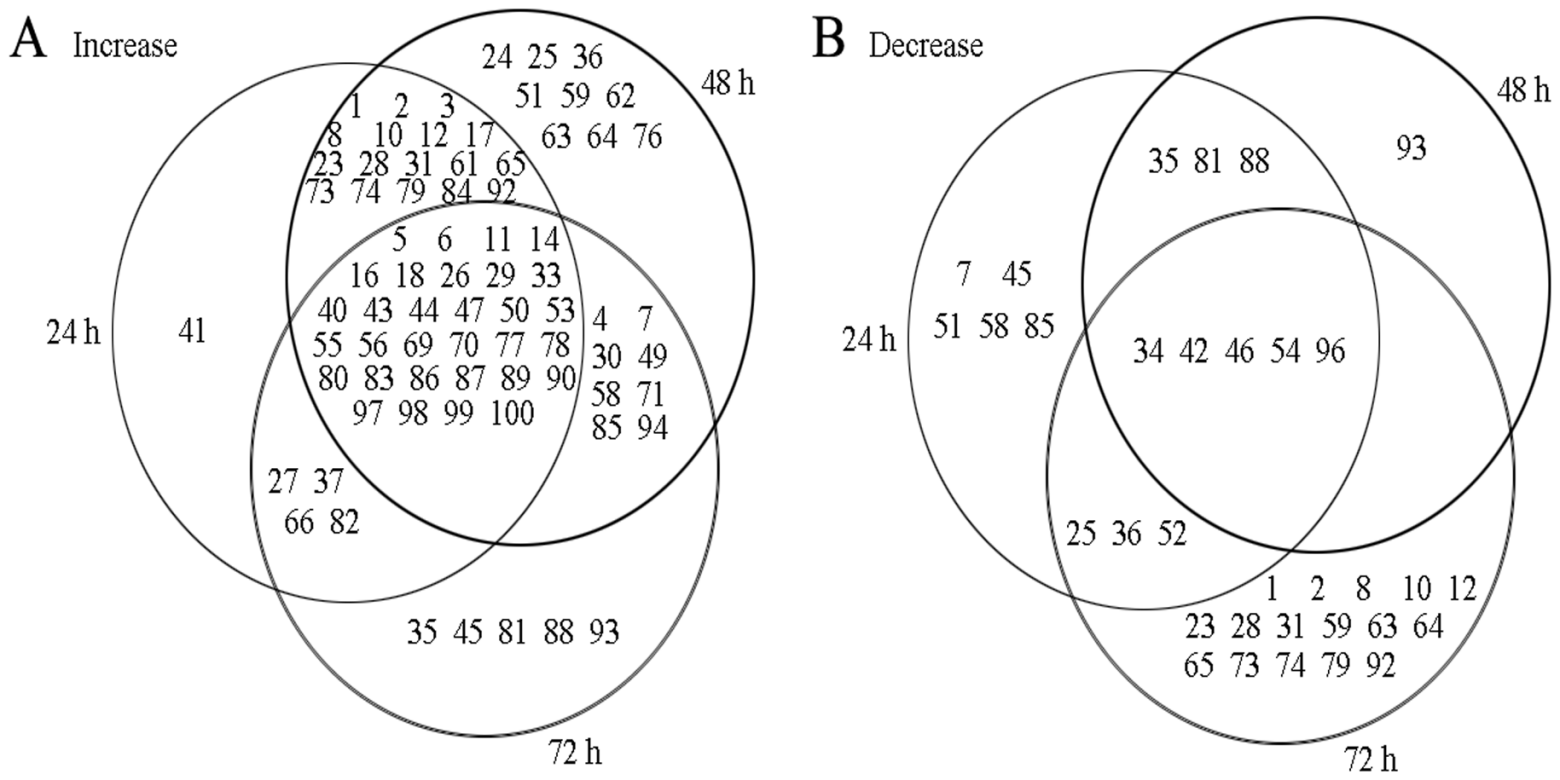
Venn diagram showing differential abundance of proteins that exhibited a significant (*P*<0.05) increase (A) or decrease (B) due to heat stress in alfalfa. Overlapping regions of the circles indicate proteins that were regulated in either the same manner in the respective treatment, whereas non-overlapping circles indicate proteins regulated in only that treatment.

**Figure 4:**
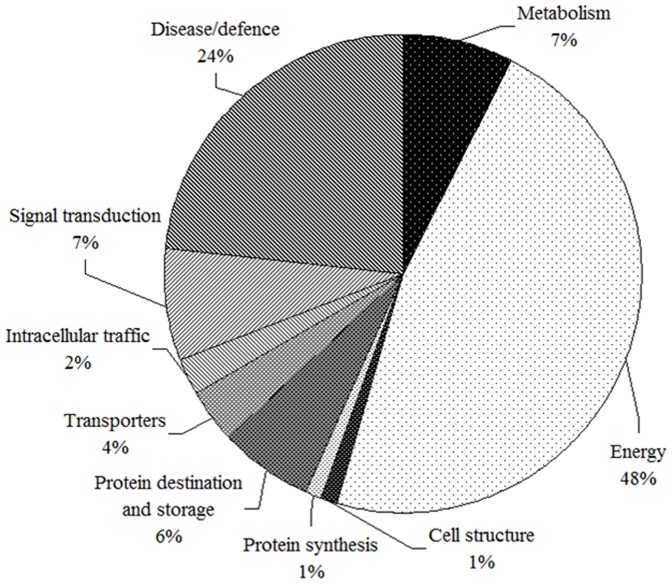
The functional category distribution of all identified proteins in response to heat stress.

81 proteins were identified by MS/MS (Table S1 and S2). The total number of proteins that exhibited either an increase or a decrease in abundance relative to their respective control line was displayed in the venn diagram (Figure 3). Among the identified protein spots, 53 spots were up-regulated and 16 spots were down-regulated at 24 h, 65 spots were up-regulated and 9 proteins were down-regulated at 48 h, 48 spots were up-regulated and 24 spots were down-regulated at 72 h. 31 spots were constantly up-regulated and 5 spots were constantly down-regulated at 24, 48 and 72 h. The identified proteins were classified according to the functional categories (Figure 4) as described by Bevan et al [19]: they belong to the categories of metabolism, energy, protein synthesis, protein destination/storage, transporters, intracellular traffic, cell structure, signal transduction and disease/defence respectively.

### Semi-quantitative Analysis of mRNA Levels

**Figure 5:**
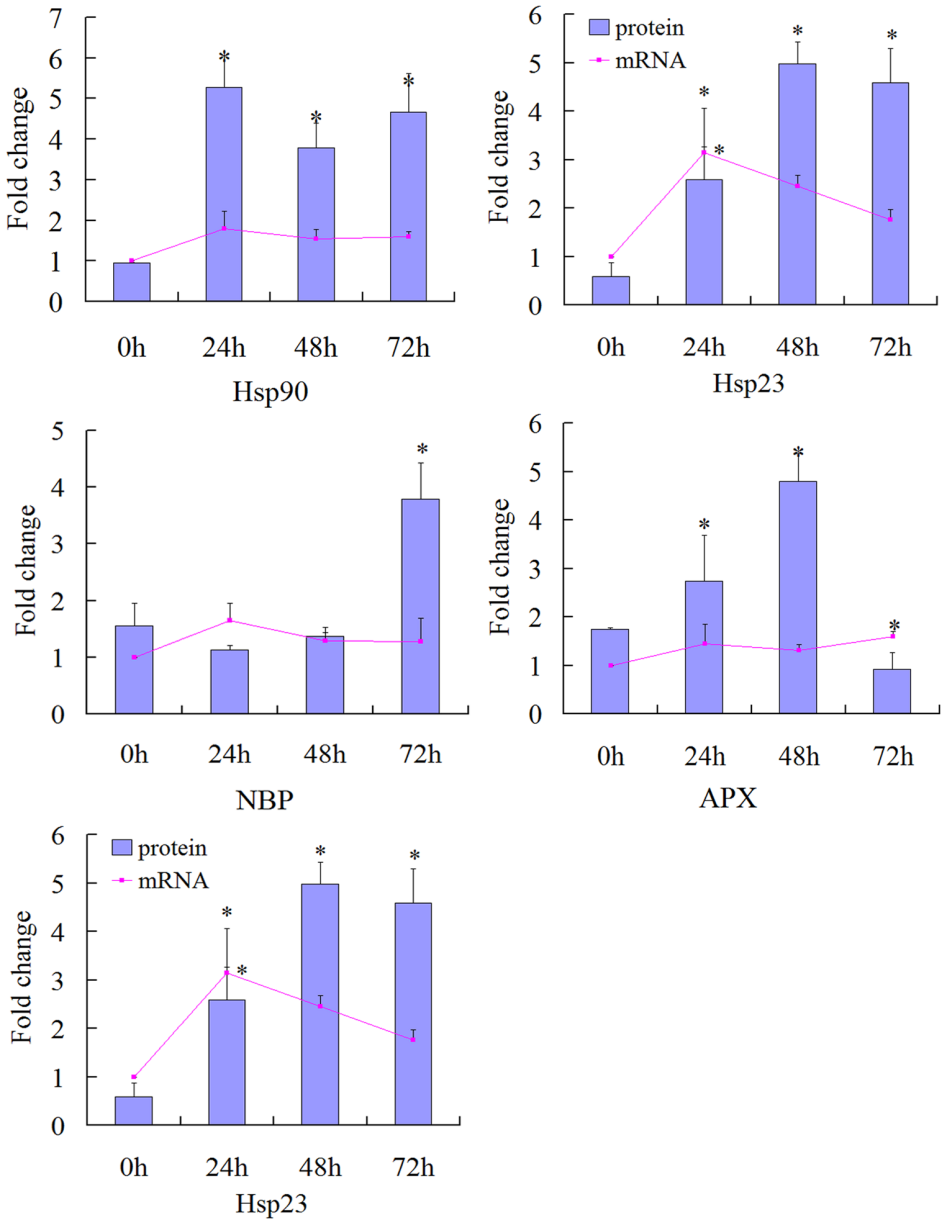
Test of the five differentially expressed proteins at mRNA levels by semi-quantitative analysis. The bars and curves represent fold changes (0, 24, 48 and 72 h at 40°C) of protein and mRNA, respectively. The asterisk shows significant differences at *P*<0.05.

Semi-quantitative analysis was used to determine the mRNA expression levels of the 5 identified proteins spots (Figure 5). 5 identified proteins were selected for analysis of expression patterns at the mRNA level in response to 40°C heat stress after treatment for 0, 24, 48 and 72 h. It was found that expressions of the Hsp 90, Hsp 17, ascorbate peroxidase (APX), Hsp 23 and nuclear acid binding protein (NBP) genes were up-regulated at 24 h and down-regulated at 48 h, Hsp 90 and APX genes were up-regulated again at 72 h while Hsp 17, Hsp 23 and NBP genes expression decreased at 72 h. These results showed that Hsp 90 presented consistent expression patterns between their protein and mRNA levels after treatment for 0, 24, 48 and 72 h at 40°C. However, Hsp 17, Hsp 23 and APX protein and mRNA showed differently expressed patterns at 48 and 72 h, while NBP expression differed at both protein and mRNA levels within all stages.

## Discussion

The overall global temperature is steadily increasing, and higher season temperatures have significant impacts on agricultural productivity. Usually, high temperature causes cell death, tissue damage and even functional disorder of the whole organism and all these changes can be further reflected in proteome alteration. In this study, we characterized the response of a stress-tolerant alfalfa plant to heat shock in order to identify stress-related proteins and understand the possible mechanisms of stress adaptation. Proteins response to heat stress are involves numerous biological processes, such as antioxidant activity, signal transduction, photosynthesis, protein destination and storage, transporters, plant defense proteins and metabolism. Of these, 17 spots were constantly up-regulated at 24 and 48 h, these proteins were mainly related to photosynthesis, ATP synthase, intracellular traffic and disease/defence, Hsp 18.2 was specially expressed at this stage. β xylosidase and ubiquitin were specially expressed at 48 h. Alcohol dehydrogenase, methyl binding domain protein and Hsp 70 were sustained expression at 48 and 72 h. 31 spots were constantly up-regulated and 5 spots were constantly down-regulated at 24 h, 48 h and 72 h. Proteins up-regulated in alfalfa were mainly enrich in energy, signal transduction, intracellular traffic and disease/defence. But the normal growth of the alfalfa was disturbed by the down-regulation of the above-mentioned biological processes at 72 h. At same time, proteins related to energy and stress protection had a stronger response in fighting against the high temperature challengers.

### Morphological and Physiological Response of Alfalfa Seedlings to Heat Stress

Transitory or constantly high temperatures cause an array of morpho-anatomical, physiological and biochemical changes in plants, which affects plant growth and development and may lead to a drastic reduction in economic yield [20]. As the heat stress time increasing, a progressive increase in plants injury, proline content and reduction in RWC were observed. Leaves were yellowed and scorched along with the declining RWC, which indicated that the physiological and biochemical processes of heat stressed plants such as photosynthetic [21] and metabolism [22] were negative affected. The decreased abundance of most Rubisco subunits and photosynthetic enzyme activity might be induced by the low RWC at 72 h. Proline was accumulated when plants were subjected from environmental stress, and the highest concentration of proline was found in growing leaves. Proline content was much lower in normal condition, but after stress proline content could accumulate to high levels by de novo synthesis or transport without inhibition any vital cellular metabolisms or enzymatic functions [23]. The increased proline content might also be induced by the up-regulation amino acid synthesis and the decreased proline content at 72 h might be due to up-regulation amino acid metabolic. This result could be identified by the increased abundance of proteasome at 72 h.

### Energy

The main effects of the heat stress were detected as changes in the abundance of proteins involved in energy pathways (photosynthesis and carbon metabolism), suggesting a close connection between these two processes and heat stress. Plants need a large quantity of ATP for sufficient energy for growth, development, and stress responses. 38 proteins in this group involved in photosynthesis, tricarboxylic acid (TCA) cycle, glycoslysis and other proteins for energy production.

23 protein spots with more than 1.5 times variation relating to photosynthesis were detected. These proteins including 4 photosystem I reaction center subunit II (PSI subunit II) (spots 1, 2, 5 and 11), 6 ribulose-1,5-bisphosphate carboxylase/oxygenase(Rubisco) large subunit (spots 26, 34, 50, 92, 99 and 100), 3 Rubisco small subunit (spots 52, 97 and 98), 3 Rubisco activase (spots 66, 80 and 85), 3 chlorophyll a/b binding protein (LHCII) (spots 29, 65 and 89), 2 oxygen-evolving enhancer protein (OEE)(spots 18 and 88), plastocyanin (spot 10) and Rubisco large subunit-binding protein β subunit (spot 83).

Photosynthesis is one of the most sensitive physiological processes to environment stressors, such as drought, salt and high temperature. Rubisco activase functions as maintenance and acclimatize photosynthetic CO_2_ fixation [24], increases the photosynthetic rate during high temperature stress and keeps the steady state of photosynthesis approached with an increase in light intensity [25]. Down-regulation of Rubisco activase might lead to short-term disruptions in CO_2_ assimilation under high temperature and they might have a low thermal stability. The up-regulation of fructose bisphosphate aldolase (FBA), glyceraldehyde-3-phosphate dehydrogenase (GADPH), Rubisco large subunit and Rubisco small subunit in heat shock may maintain the CO_2_ assimilation rate. The increased abundance of Rubisco activase at 48 and 72 h could enhance the photosynthetic activity [26]. Rubisco catalyzes the first step of carbon fixation, so the up-regulated Rubisco proteins may enhance photosynthesis. PSI subunit II can serve as a template around which other PSI reaction center subunits assemble and up-regulation of this protein may enhance the stability of PSI to heat stress. Plastocyanin is an essential protein and donates electrons to PSI, the up-regulation of this protein could increase the total rate of energy transduction in a photosynthetic membrane. The down-regulation of PSI subunit II and plastocyanin indicate the structure damage of PSI and reduction of electron-transfer would happen at 72 h. LHCII represents a system for balancing the excitation energy between the two photosystems [27]. The enhanced resistance of PSI associated with the induction of LHCII. Some up-regulated proteins related to photosynthesis were also detected in this study, such as OEE were also detected in this study. The expression of OEE family genes is related to abiotic stresses such as cold stress [8] and heat stress [16]. But in other species, this protein was negative affected by several environment stresses [28], [29].

In addition to photosynthesis, several proteins involved in energy production were identified. These include 5 proteins involved in the TCA cycle; including citrate synthase, oxoglutarate dehydrogenase, fumarate hydratase and malate dehydrogenase. 5 proteins involved in glycoslysis including FBA, GADPH and enolase. Some other proteins related to energy production, including nucleoside diphosphate kinse, cupin domain containing protein, NAD-dependent dehydrogenase, ATP synthase β subunit and ATP synthase CF1 β subunit. Citrate synthase and oxoglutarate dehydrogenase were the key enzymes in TCA cycle and the up-regulation of their abundance could speed up the energy metabolism and provide more energy for heat stress resistance. 5 proteins involved in glycoslysis were down-regulated at different thermal stage, which reduces the synthesizing of carbon skeleton provide for other pathway [30]. Increased accumulation of GAPDH implies that more 3-phosphoglycerate was formed. 3-phosphoglycerate is a pinitol precursor and pinitol may act as an osmolyte. Cupin domain containing protein, ATP synthase β subunit and ATP synthase CF1 β subunit were increased at 24 and 48 h. The β subunit of ATP synthase is the primary catalytic site for ATP synthase. Nucleoside diphosphate kinse uses ATP to maintain cellular levels of CTP, GTP, and UTP and can enhance multiple stress tolerance in transgenic plants [31]. Cupin domain containing protein has biochemical activities associated with the cell wall, this protein have the function to protect other proteins against thermal denaturation, one of the cupin proteins in cultured shoots of aspen was found exposed to water stress [32]. The down-regulation of fumarate hydratase, malate dehydrogenase and oxoglutarate dehydrogenase content indicated that the inhibition of energy metabolism happened at 72 h, suggesting that high temperature might reduce carbohydrate metabolism that further affect energy-dependent processes involved in heat stress resistance.

### Metabolism

There were 6 proteins (spots 42, 56, 58, 62, 63 and 86) detected that are associated with plant metabolism. Protein quality-control system may play an important role in the production of osmotic regulators. The improved metabolism of nitrogen, sugars and sugar alcohols may be an important tolerance mechanism induced by high temperature. Aspartate aminotransferase and glutamine synthetase (GS) were up-regulated in all thermal stages, alcohol dehydrogenase, formate—tetrahydrofolate ligase and β xylosidase were only up-regulated at 48 h, while glutamate 1-semialdehyde aminotransferase was down-regulated in all thermal stages. Aspartate aminotransferase and GS play an important role in regulating nitrogen metabolism in almost all organisms and the decline in the activity of aspartate aminotransferase influences various aspects of nitrogen metabolism in drought-stressed rice [33]. GS is the key enzyme involved in the assimilation of ammonia derived either from nitrate reduction, N_2_ fixation and photorespiration or asparagine breakdown. Overexpression of cytosolic GS enhanced photorespiration and could contribute to the protection of photosynthesis under stress condition [34]. Increases in GS activities are also found in plant under salt [35] and drought stress [36]. In plants, alcohol dehydrogenase converts acetaldehyde to ethanol, with the concomitant regeneration of NAD^+^ for glycolysis which carrying electrons from one reaction to another, and also used in other cellular processes. It is the most notable substrate of enzymes that add or remove chemical groups from proteins, in posttranslational modifications. The increased NAD^+^ could enhance redox reactions, the expression of alcohol dehydrogenase and its contribution to the plant's ability to survive might depend on the carbon flow and ethanol fermentation of the plant species. The abundance of alcohol dehydrogenase also increases in the presence of lack of oxygen [37]. β xylosidase is involved in cell wall polysaccharide disassembly or modification and may function in cell wall degradation [38]. The up-regulation of this protein indicates that damage to the cell wall would happen at 48 h. Formate—tetrahydrofolate ligase is one of the enzymes participating in the transfer of one-carbon units, an essential element of various biosynthetic pathways, which was also essential for the synthesis of purines, thymidylate, methionine and formylmethionyl-tRNA. Thus is central to de novo purine and amino acid biosynthesis, up-regulated of this protein indicates that plays a major role under high temperature conditions. This protein was also decreased in a low oxygen environment [39], this indicated that oxygen deficit would happen at 24 and 72 h. Glutamate 1-semialdehyde aminotransferase catalyzes the last step in the sequential conversion of glutamate to 5-aminolevulinate and protects for chlorophyll biosynthesis. Decreased 5-aminolevulinate synthesis would result in deprivation of the overall capacity of chlorophyll formation. This enzyme acts in the greening process in stressful environment and also elevated in heat-stressed wheat seedlings [40].

### Protein Synthesis, Protein Destination and Storage and Transporters

There were 9 proteins (spots 3, 4, 23, 33, 47, 53, 70, 87 and 94) belonging to these categories. Among these proteins, the abundance of proteasome (spot 23) decreased at 72 h. Proteasome and cysteine proteinase can degrade unneeded or damaged proteins by proteolysis. Proteasome mediated proteolysis has been shown to be an important aspect of low salt stress response [41]. The regulation of the heat stress response may also be dependent on the cellular control of degradation and the quality maintenance of proteomes. Proteins involved in the ubiquitin–proteasome pathway appear to be involved in selective protein degradation during senescence [42]. Eukaryotic translation initiation factor 3 subunit I is a component of eIF3 that was involved in mRNA translation [43]. Eukaryotic translation initiation factor 3 subunit I positively regulated cell cycle progression, proliferation and the signal-regulated kinase pathway which is involved in many fundamental cellular processes [44], but negatively regulated cell motility and invasion. This activity of eukaryotic translation initiation factor 3 subunit I might be due to its recruitment to stress granules and/or its ability to differentially regulate mRNA translation during cellular stress. Binding protein is implicated in cotranslational folding of nascent polypeptides, and in the recognition and disposal of misfolded polypeptides [45]. Peptidyl-prolyl cis-trans isomerase and protein disulfide isomerase act to catalyze protein folding. Peptidyl-prolyl cis-trans isomerase could accelerate the folding of proteins [46] and protein disulfide isomerase is involved to the posttranslational modification disulfide exchange [47]. The up-regulation of these proteins could accelerate the protein synthesis and have a positive effect on heat tolerance. Porins are voltage-gated diffusion pores found in all eukaryotic kingdoms that have the function of diffusing small hydrophilic nutrients and waste products. The dynamic endomembrane system of eukaryotic cells is emerging as a critical and central coordinator in cell development, growth, signaling, and adaptation to stress [48].

### Intracellular Traffic, Cell Structure and Signal Transduction

Intracellular traffic and signal transduction are important in many different aspects of cellular activity. In this catalog, 2 isoflavone reductase-like NAD(P)H-dependent oxidoreductase (IFR) were down-regulated at different stages, nuclear acid binding protein was down-regulated at 24 h, Tic40 and profilin were down-regulated at 72 h. Most of this part protein was up-regulated under heat stress and this indicated enhanced intracellular traffic, cell stability and signal transduction processes. Under high temperature stress, the net loss in photosynthetic function could be due to degradation of chloroplast proteins coupled with impaired posttranslational targeting of their precursor proteins into the chloroplast [49]. Toc75 and Tic40 are associated with import machinery [50], and have the function of importing a variety of preproteins during translocation across the inner envelope membrane. Toc75 most likely functions as the protein-translocating channel in the outer membrane. Tic40 functions as a co-chaperone in the stromal chaperone complex that facilitates protein translocation across the inner membrane [51], which also have the capable of adjusting their influence and physical associations during temperature stress. Profilin (12–15 kDa) is involved in the dynamic turnover and restructuring of the actin cytoskeleton and spatially and temporally controlled growth of actin microfilaments, which is an essential process in cellular locomotion and cell shape changes. Profilin plays a role in maintaining normal F-actin levels in response to shifts to high temperature [52]. Caffeic acid 3-O-methyltransferase may induce lignin biosynthesis and lignin biosynthesis, lignin biosynthesis is also induced by wounding and metabolic stress [53], [54]. The increased profilin and lignin has a protection role in the cytoskeleton and cell wall. IFR is involved in the biosynthesis of isoflavonoid phytoalexins in legumes and several IFRs also responsed to biotic or abiotic stress [55], [56]. Methyl binding domain proteins are used to determine the methylation state, which has been investigated extensively as part of the mechanism of gene silencing and with regard to their modulation of other chromatin functions [57]. Loss of DNA methylation reduces the ability of Arabidopsis plants to tolerate salt stress conditions [58]. DNA-binding proteins could protect DNA, the up-regulation of this protein was also founded during oxidative stress [59] and abscisic acid stress [60].

### Disease/Defence

19 of the identified proteins belong to this group. All the proteins in this group were up-regulated for at least one time stage. This implies that these proteins are associated mainly with the enhanced heat tolerance. In recent study, a total of 7 proteins were identified as Hsp 90, Hsp 70 and sHsps. 5 of them were sHsps (Hsp 17, Hsp 18.2, Hsp 20 and two Hsp 23), which were the most ubiquitous Hsps subgroup [61]. Hsps are chaperones and play a crucial role in protecting plants against stress by re-establishing normal protein conformation and thus cellular homeostasis. They can assist in protein refolding under heat stress conditions. Hsp 90 and Hsp 70 were up-regulated all the heat stressed stage. Expression of Arabidopsis Hsp 90 is developmentally regulated and responds to heat, cold, salt stress, heavy metals, phytohormones and light and dark transitions [62]. The overexpression of Hsp 70 genes were correlated positively with the acquisition of thermotolerance [63] and resulted in enhancing the tolerance of high-temperature stress in plants [64]. Spots 6, 12, 14, 24, 30 were identified as sHsps. The high diversification of plant sHsps probably reflects a molecular adaptation to stress conditions that are unique to plants [65]. Plant sHsps are all nuclear-encoded and divided into 6 classes: 3 classes of (classes CI, CII and CIII) of sHsps are localized in the cytosole or in the nucleus and the other three in the plastids, the endoplasmic reticulum and the mitochondria (CIV, CV and CVI). 2 spots (12 and 30) were identified as cytoplasm-targeted sHsps, whereas spots 14 and 24 were identified as chloroplast-targeted sHsps, and spot 6 was identified as a nucleus-located sHsps. The up-regulation or induction of sHsps has also been noticed in several heat stress related proteomic analyses, which suggested that sHsps may be used as a biomarker for heat stress [16], [66]. Together with these reports, the recent results provided a clear evidence indication that sHsps play an important role in plant-acquired heat stress tolerance.

Beside Hsps, other proteins were also detected in this experiment, such as ascorbate peroxidase, glucan endo-1,3-β-glucosidase, thaumatin-like protein, ubiquitin, dehydroascorbate reductase, 20 kDa chaperonin, germin-like protein and mitochondrial peroxiredoxin. Oxidative stress producing reactive oxygen species is a major component of heat stress [67]. APX is the main enzyme responsible for hydrogen peroxide removal in the chloroplasts and cytosol of higher plants, APX detoxify peroxides such as hydrogen peroxide using ascorbate as a substrate [68]. Ascorbic acid is a strong antioxidant that is effective in scavenging superoxide (O^2-^'), hydroxyl (OH') radicals and singlet oxygen. It can also remove H_2_O_2_. Dehydroascorbate reductase are key enzymes of the ascorbate-glutathione cycle that maintain reduced pools of ascorbic acid and serve as important antioxidants [69], some of these enzymes may have the thermal stability [70]. Kuo identified chloroplast-localized co-chaperonin chaperonin 20 as a mediator of FeSOD activation by direct interaction. Chaperonin 20 alone could enhance SOD1, SOD2 and SOD3 activity [71]. Plant β-1, 3-glucanases are important in the defense of plants against pathogen attack and implicated in diverse physiological and developmental processes, which are induced in response to biotic or abiotic stress [72], and that could degrade the cell wall of pathogen and indirectly promote the release of cell-wall derived materials that can act as elicitors in defensive reactions. Thaumatin-like protein is known to protect plants against biotic and abiotic stresses. Thaumatin-like protein is stable at low pH conditions and resistant to heat treatment and proteolytic degradation [73]. The up-regulation of this protein was also found in heat stressed Arabidopsis [74]. Plant growth and development are largely influenced by ubiquitin-mediated regulation of protein stability and ubiquitin conjugation as a major regulator of stress-responsive transcription factors and other regulatory proteins under stress condition. Ubiquitin mediated proteolysis may protect organisms from heat stress by removing improperly functioning proteins [75]. Ubiquitin and conjugated-ubiquitin act as an important mechanism of heat tolerance in *Prosopis chilensis* and *Glycine max* under heat stress [76]. Germin-like protein plays an important role in response to oxidative stress and expressed during specific periods of plant growth and development, a pattern of evolutionary subfunctionalization. Peroxiredoxins are abundant antioxidant enzymes that use an active site cysteine to catalyse the reduction of peroxides [77]. Peroxiredoxins also function as molecular chaperones [78], and their abundance and reactivity makes them ideal peroxide sensors that could facilitate the oxidation of less reactive thiol proteins [79]. These results suggest that high temperature enhanced the antioxidant defense system to decrease oxidative damage under heat stress, possibly providing a favorable environment for growth and development processes. Hsps were also expressed for protecting plants against stress by re-establishing normal protein conformation and thus cellular homeostasis.

### mRNA Levels of Gene Expression

Semi-quantitative analysis was used to investigate changes in expression patterns at the mRNA level. Among all identified proteins, NBP, APX, Hsp 17, Hsp 23 and Hsp 90 were affected in response to heat stress at the mRNA levels. At the proteomic level, these proteins showed an increasing pattern for at least one time point. Hsp 90 exhibited a consistent level of expression between the protein and mRNA levels; However, the mRNA levels of Hsp 17, Hsp 23 and APX showed consistent between transcriptional and translational levels at first 24 h. Excessive exposure to high temperature would lead to gene or protein degradation, these genes may have the adaptation ability to high temperature. The different expression from those genes to the corresponding proteins was showed at 48 and 72 h, this might be due to the differential endurance to high temperature. The NBP gene showed differential patterns between protein and mRNA levels. This result was supported by the previously established concept that transcription patterns are not directly concomitant with protein expression levels [80]. The discrepancies observed between mRNA and protein abundance might also due to the time shift observed between the detection of the transcript and its cognate protein.

## Conclusions

In summary, the analyses of the phenotypes, RWC, proline content and leaf proteome that was conducted in this work provides new insights to the effects of high temperature on alfalfa. The significantly decreased RWC and increased proline content in alfalfa leaves were found after heat stress process. High temperature also triggered a significant proteome variation of alfalfa. Although several proteomic analyses have been carried out to analyze alfalfa-leaf proteome expression under drought and wet, to our knowledge, there have not been any reports regarding to proteomic analyses of heat stress. Thus, proteomic profiling is imperative in order to analyze the heat-stress responsive proteins in alfalfa leaves against the thermal defense in this heat tolerance plant. There are 81 differentially expressed proteins were identified under high temperature stress using 2-DE and MS based proteomic approaches. The proteins identified were implicated in a wide range of biological processes with the majority having a putative role in energy production, catabolism, remobilization, stress and defense responses. These defense response proteins were involved in multiple processes such as redox homeostasis regulation, heat stress response and detoxify peroxides. The protein disease/defence category contained a large set of chaperone proteins and proteins involved in disulfide bond regulation. These proteins may also contribute to better thermo-tolerance in alfalfa. The function of genes encoding these differentially expression proteins among the different heat stress times may be further investigated using molecular approaches, which may provide the molecular basis of the thermo-tolerance in alfalfa.

## Supporting Information

Table S1 Differentially expressed proteins identified by mass spectrometry between 24, 48 and 72 h heat stress (40°C) and normal temperature (25°C).
(DOC)

Table S2 The homologs of unknown and hypothetical proteins identified by mass spectrometry between 24, 48 and 72 h heat stress (40°C) and normal temperature (25°C).
(DOC)
