# Supplementary material for: Proteomics Analysis of Alfalfa Response to Heat Stress
Source: PLoS One. 2013 Dec 6;8(12):e82725. doi: 10.1371/journal.pone.0082725 (PMC3855785; doi:10.1371/journal.pone.0082725)
Supplement: Table S1 — Differentially expressed proteins identified by mass spectrometry between 24, 48 and 72 h heat stress (40°C) and normal temperature (25°C). (DOC) [file pone.0082725.s001.doc]

Table 1 Differentially expressed proteins identified by mass spectrometry between 24, 48 and 72 h heat stress (40°C) and normal temperature (25°C)

| **Spot no.** | **MOWSE score** | **PM** | **Theoretical(Mr/ pI)** | **Observed(Mr/ pI)** | **Protein name/Species/Acc.no.** | **Regulation** | | | |
| --- | --- | --- | --- | --- | --- | --- | --- | --- | --- |
|  |  |  |  |  |  | **Control** | **24 h** | **48 h** | **72 h** |
| **01 Metabolism** | | | | | | | | | |
| 58 | 110 | 6 | 40.6/6.14 | 56/6.8 | Alcohol dehydrogenase class/Medicago truncatula/gi|357510277 | 167.86 | 107.4 ^a^ | 452.63 ^a^ | 235.4 ^a^ |
| 56 | 161 | 14 | 47.2/8.13 | 51/7.1 | Aspartate aminotransferase/Medicago truncatula/gi|357474641 | 71.8 | 152.83 ^a^ | 312.06 ^a^ | 297.2 ^a^ |
| 63 | 542 | 19 | 82.5/8.76 | 80/8.5 | Beta xylosidase/Medicago truncatula/gi|357449039 | 331.8 | 346.9 | 719.4 ^a^ | 195.86 ^a^ |
| 62 | 176 | 8 | 79.6/7.09 | 83/7 | Formate-tetrahydrofolate ligase/Medicago truncatula/gi|357445035 | 244.46 | 279.8 | 531.06 a | 301.63 |
| 42 | 434 | 18 | 49.9/6.36 | 53/6 | Glutamate 1-semialdehyde aminotransferase/Medicago sativa /gi|345451030 | 559.4 | 213.6 ^a^ | 139.86 ^a^ | 0 ^a^ |
| 86 | 269 | 14 | 47.0/6.29 | 54/5.3 | Glutamine synthetase/Medicago truncatula/gi|28629470 | 960.1 | 1448.7 ^a^ | 1815.93 ^a^ | 2526.23 ^a^ |
| **02 Energy** | | | | | | | | | |
| 79 | 314 | 15 | 52.7/5.27 | 66/5.3 | ATP synthase CF1 beta subunit/Medicago truncatula/gi|153012228 | 438.3 | 2695.93 ^a^ | 606.6 ^a^ | 229.8 ^a^ |
| 84 | 1100 | 37 | 52.6/5.34 | 64/5.3 | ATP synthase subunit beta/Medicago truncatula/gi|358349385 | 1145.46 | 1944.26 ^a^ | 1463.16 ^a^ | 1185.16 |
| 29 | 101 | 4 | 28.2/5.47 | 32/5.4 | Chlorophyll a/b binding protein/Medicago sativa/gi|3293555 | 1311.13 | 3521.56 ^a^ | 2472.1 ^a^ | 2819.5 ^a^ |
| 65 | 124 | 4 | 28.7/5.29 | 31/5.2 | Chlorophyll a/b binding protein/Medicago truncatula/gi|357495319 | 1143.73 | 1642.33 ^a^ | 2882.7 ^a^ | 550.9 ^a^ |
| 89 | 103 | 7 | 28.7/5.29 | 31/5 | Chlorophyll a/b binding protein/Medicago truncatula/gi|357495319 | 561.1 | 743.43 ^a^ | 1566.96 ^a^ | 1082.76 ^a^ |
| 44 | 93 | 9 | 52.3/8.52 | 55/6.5 | Citrate synthase/Glycine max/gi|366985125 | 442 | 675.46 ^a^ | 643.43 ^a^ | 697.83 ^a^ |
| 73 | 103 | 9 | 49.5/9.16 | 62/6 | Dihydrolipoyllysine-residue succinyltransferase component of 2-oxoglutarate dehydrogenase complex/Medicago truncatula /gi|357445475 | 89.96 | 131.2 ^a^ | 216.1 ^a^ | 39 ^a^ |
| 46 | 586 | 13 | 47.6/5.31 | 64/5.6 | Enolase/Glycine max/gi|351724891 | 1280.63 | 604.66 ^a^ | 709.2 ^a^ | 716.3 ^a^ |
| 41 | 632 | 19 | 42.9/6.39 | 43/5.7 | Fructose bisphosphate aldolase/Medicago sativa/gi|227464396 | 1324.4 | 2351.43 ^a^ | 1062.76 | 1229.13 |
| 51 | 362 | 18 | 78.3/5.76 | 46/6.8 | Fructose-bisphosphate aldolase/Medicago truncatula/gi|357490465 | 1308.1 | 850.4 ^a^ | 1996.83 ^a^ | 1211.83 |
| 59 | 114 | 6 | 53.2/8.72 | 61/7.5 | Fumarate hydratase/Medicago truncatula/gi|357442389 | 163 | 146.4 | 335.3 ^a^ | 94.76 ^a^ |
| 35 | 512 | 18 | 36.6/6.55 | 45/7.4 | Glyceraldehyde-3-phosphate dehydrogenase/Medicago truncatula /gi|357477179 | 1980.56 | 1114.93 ^a^ | 1200.7 ^a^ | 2613.06 ^a^ |
| 36 | 279 | 6 | 35.8/8.8 | 45/6.2 | Malate dehydrogenase precursor/Medicago sativa/gi|2827080 | 786.96 | 310.2 ^a^ | 1633.56 ^a^ | 0 ^a^ |
| 81 | 342 | 8 | 35.8/8.8 | 45/6.4 | Malate dehydrogenase precursor/Medicago sativa/gi|2827080 | 547.16 | 0 ^a^ | 360.1 ^a^ | 1974.26 ^a^ |
| 7 | 139 | 6 | 25.2/9.37 | 17/6.1 | Nucleoside diphosphate kinase 3/Medicago truncatula/gi|226088577 | 489.36 | 250.83 ^a^ | 735.23 ^a^ | 662.13 ^a^ |
| 18 | 409 | 10 | 29.1/9.08 | 27/7.1 | Oxygen-evolving enhancer protein/Medicago truncatula /gi|357494079 | 582.53 | 2263.23 ^a^ | 4397.83 ^a^ | 2974.2 ^a^ |
| 88 | 549 | 13 | 34.9/6.25 | 37/5.2 | Oxygen-evolving enhancer protein/Medicago truncatula /gi|358344003 | 1456.5 | 778.13 ^a^ | 3507.73 ^a^ | 2777.83 ^a^ |
| 1 | 521 | 17 | 23.0/9.58 | 25/8.9 | Photosystem I reaction center subunit II/Medicago truncatula /gi|357480841 | 1247.367 | 3917.43 ^a^ | 2344.3 ^a^ | 0 ^a^ |
| 2 | 457 | 8 | 23.0/9.58 | 25/8.4 | Photosystem I reaction center subunit II/Medicago truncatula /gi|357480841 | 241.1 | 2063.23 ^a^ | 1325.3 ^a^ | 0 ^a^ |
| 5 | 349 | 12 | 23.0/9.58 | 20/6.8 | Photosystem I reaction center subunit II/Medicago truncatula /gi|357480841 | 1607.46 | 2484.13 ^a^ | 4293.53 ^a^ | 4433.5 ^a^ |
| 11 | 393 | 12 | 23.0/9.58 | 18/5.4 | Photosystem I reaction center subunit II/Medicago truncatula /gi|357480841 | 795.73 | 1244.53 ^a^ | 999.83 ^a^ | 1016.43 ^a^ |
| 10 | 112 | 5 | 17.0/4.99 | 15.5/4.5 | Plastocyanin/Medicago truncatula/gi|357467117 | 1369.46 | 3190.46 ^a^ | 2965.4 ^a^ | 0 ^a^ |
| 50 | 245 | 9 | 50.2/6.22 | 90/6 | Ribulose 1,5-biphosphate carboxylase large subunit/Medicago sativa /gi|1223773 | 370.33 | 1211.46 ^a^ | 960.06 ^a^ | 1817.93 ^a^ |
| 52 | 206 | 7 | 20.0/8.86 | 15.6/6.3 | Ribulose-1,5-bisphosphate carboxylase small subunit/Medicago sativa /gi|16224234 | 7705.46 | 1939.16 ^a^ | 7351.2 | 5737.63 ^a^ |
| 66 | 392 | 12 | 52.2/6.1 | 60/5.4 | Ribulose-1 5-bisphosphate carboxylase/oxygenase activase /Medicago truncatula /gi|357492323 | 1142.43 | 4040.2 ^a^ | 1033.73 | 1835.3 ^a^ |
| 80 | 609 | 18 | 52.2/6.1 | 60/5.3 | Ribulose-1 5-bisphosphate carboxylase/oxygenase activase /Medicago truncatula /gi|357492323 | 580.5 | 1060.26 ^a^ | 3935.5 ^a^ | 877.03 ^a^ |
| 85 | 426 | 12 | 52.2/6.1 | 58/5.2 | Ribulose-1 5-bisphosphate carboxylase/oxygenase activase /Medicago truncatula /gi|357492323 | 373.16 | 0 ^a^ | 1102.63 ^a^ | 2368.46 ^a^ |
| 97 | 295 | 16 | 119.0/6.15 | 16/6 | Ribulose-1,5-bisphosphate carboxylase small subunit/Medicago sativa /gi|16224234 | 335.33 | 1385.56 ^a^ | 921.46 ^a^ | 4345.5 ^a^ |
| 98 | 363 | 15 | 119.0/6.15 | 15/6.2 | Ribulose-1,5-bisphosphate carboxylase small subunit/Medicago sativa /gi|16224234 | 649.2 | 1429.53 ^a^ | 778.86 ^a^ | 1635.83 ^a^ |
| 26 | 177 | 9 | 51.9/6.12 | 27/5.2 | Ribulose-1,5-bisphosphate carboxylase/oxygenase large subunit /Burchellia bubaline /gi|1769935 | 543.53 | 1634.86 ^a^ | 1018.93 ^a^ | 1943.8 ^a^ |
| 34 | 86 | 6 | 25.5/6 | 45/7.3 | Ribulose-1,5-bisphosphate carboxylase/oxygenase large subunit /Medicago sativa/gi|340511632 | 3275.53 | 913.03 ^a^ | 1703.06 ^a^ | 1250.6 ^a^ |
| 92 | 252 | 6 | 22.3/5.76 | 18/4.6 | Ribulose-1,5-bisphosphate carboxylase/oxygenase large subunit /Medicago truncatula/gi|313664305 | 1918.6 | 3025.1 ^a^ | 4402.83 ^a^ | 0 ^a^ |
| 99 | 89 | 7 | 51.9/6.75 | 14.5/6.5 | Ribulose-1,5-bisphosphate carboxylase/oxygenase large subunit /Delonix brachycarpa/gi|66735845 | 0 | 161.8 ^a^ | 438.3 ^a^ | 186.4 ^a^ |
| 100 | 141 | 9 | 51.9/6.75 | 14.5/6.8 | Ribulose-1,5-bisphosphate carboxylase/oxygenase large subunit /Delonix brachycarpa/gi|66735845 | 360.3 | 1602.1 ^a^ | 729.63 ^a^ | 3288.9 ^a^ |
| 83 | 422 | 13 | 62.8/5.72 | 82/5.3 | RuBisCO large subunit-binding protein subunit beta, chloroplastic-like /Glycine max/gi|356556926 | 332.4 | 927.2 ^a^ | 616.66 ^a^ | 643.1 ^a^ |
| **05 Protein synthesis** | | | | | | | | | |
| 33 | 181 | 8 | 35.2/6.83 | 44/7.2 | Eukaryotic translation initiation factor 3 subunit I/Medicago truncatula /gi|357453275 | 111.83 | 182.53 ^a^ | 248.26 ^a^ | 241.1 ^a^ |
| **06 Protein destination and storage** | | | | | | | | | |
| 70 | 148 | 10 | 73.2/5.11 | 95/5.4 | BiP isoform A/Glycine max/gi|351722422 | 594.1 | 2304.9 ^a^ | 2108.3 ^a^ | 2412.86 ^a^ |
| 87 | 149 | 7 | 31.9/4.9 | 38/5.1 | Cysteine proteinase/Medicago truncatula/gi|357437721 | 138.26 | 257.7 ^a^ | 420.76 ^a^ | 409.4 ^a^ |
| 3 | 199 | 7 | 18.1/8.36 | 19/8.1 | Peptidyl-prolyl cis-trans isomerase/Medicago truncatula /gi|357473975 | 4399.46 | 5850.76 ^a^ | 8289.73 ^a^ | 4878.23 |
| 4 | 237 | 6 | 18.1/8.36 | 19/7.6 | Peptidyl-prolyl cis-trans isomerase/Medicago truncatula /gi|357473975 | 1171.13 | 1334.9 | 2504.06 ^a^ | 2953.53 ^a^ |
| 23 | 90 | 4 | 24.8/5.72 | 26/5.5 | Proteasome subunit beta type/Medicago truncatula/gi|357466571 | 36.66 | 256.2 ^a^ | 305.23 ^a^ | 0 ^a^ |
| **07 Transporters** | | | | | | | | | |
| 94 | 470 | 15 | 29.6/8.91 | 35/9.4 | Outer plastidial membrane protein porin/Medicago truncatula /gi|357501641 | 990.73 | 1252.66 | 1871.3 ^a^ | 2490.8 ^a^ |
| **08 Intracellular traffic** | | | | | | | | | |
| 61 | 260 | 25 | 88.0/6.99 | 98/7 | Protein TOC75/Medicago truncatula/gi|357472869 | 93.26 | 136.43 ^a^ | 229.33 ^a^ | 121.03 |
| 74 | 93 | 2 | 47.1/6.18 | 52/5 | Translocon Tic40/Pisum sativum/gi|5531416 | 307.6 | 579.53 ^a^ | 572.33 ^a^ | 181.83 ^a^ |
| **09 Cell structure** | | | | | | | | | |
| 8 | 116 | 8 | 14.1/4.87 | 15/5.3 | Profilin /Medicago truncatula/gi|357520819 | 929.3 | 1401.53 ^a^ | 1688.96 ^a^ | 0 ^a^ |
| **10 Signal transduction** | | | | | | | | | |
| 43 | 170 | 7 | 40.0/5.8 | 50/6.1 | Caffeic acid 3-O-methyltransferase/Medicago truncatula /gi|357470921 | 147.03 | 678.13 ^a^ | 543.5 ^a^ | 763.23 ^a^ |
| 37 | 283 | 13 | 33.9/6.46 | 44/6.3 | Isoflavone reductase-like NAD(P)H-dependent oxidoreductase /Medicago sativa/gi|6525021 | 1496.8 | 3275.1 ^a^ | 1677.06 | 2570.13 ^a^ |
| 64 | 431 | 15 | 33.9/6.46 | 44/6.5 | Isoflavone reductase-like NAD(P)H-dependent oxidoreductase /Medicago sativa/gi|6525021 | 590.46 | 544.46 | 1136.76 ^a^ | 0 ^a^ |
| 96 | 211 | 8 | 33.9/6.46 | 49/6.3 | Isoflavone reductase-like NAD(P)H-dependent oxidoreductase /Medicago sativa/gi|6525021 | 861.53 | 0 ^a^ | 236.9 ^a^ | 0 ^a^ |
| 49 | 140 | 8 | 38.8/4.66 | 81/4.9 | Methyl binding domain protein/Medicago truncatula/gi|357478139 | 1198.93 | 1498.76 | 2265.76 ^a^ | 2746.93 ^a^ |
| 45 | 136 | 2 | 45.5/6.31 | 60/6.5 | Putative nuclear acid binding protein/Medicago sativa/gi|365192966 | 312.36 | 225.26 ^a^ | 273.7 | 756.96 ^a^ |
| **11 Disease/defence** | | | | | | | | | |
| 6 | 216 | 10 | 17.9/7.74 | 18/6.4 | 17kD heat shock protein/Medicago truncatula/gi|1552553 | 365.6 | 1480.86 ^a^ | 1456.16 ^a^ | 1677.86 ^a^ |
| 12 | 351 | 14 | 18.1/5.81 | 20/5.4 | 18.2 kDa class I heat shock protein/Medicago truncatula /gi|357475983 | 609.6 | 6234.2 ^a^ | 8464.56 ^a^ | 0 ^a^ |
| 30 | 127 | 8 | 21.7/5.81 | 30/5.9 | 20 kDa chaperonin/Medicago truncatula/gi|357479373 | 550.9 | 623.3 | 1136.03 ^a^ | 1586.4 ^a^ |
| 27 | 375 | 11 | 20.1/5.33 | 27/5.3 | Ascorbate peroxidase/Medicago sativa/gi|16304410 | 649.7 | 1077 ^a^ | 563 | 890.33 ^a^ |
| 28 | 362 | 5 | 20.1/5.33 | 32/5.3 | Ascorbate peroxidase/Medicago sativa/gi|16304410 | 1043.73 | 1642.33 ^a^ | 2882.7 ^a^ | 550.83 ^a^ |
| 82 | 489 | 19 | 20.1/5.33 | 31/5.2 | Ascorbate peroxidase/Medicago sativa/gi|16304410 | 995.86 | 2603.76 ^a^ | 1249.23 | 1554.7 ^a^ |
| 54 | 455 | 16 | 40.3/8.79 | 36/7.7 | Glucan endo-1,3-beta-glucosidase/Medicago truncatula /gi|357448997 | 2185.7 | 736.7 ^a^ | 1159.96 ^a^ | 238.66 ^a^ |
| 71 | 384 | 24 | 72.3/5.53 | 83/5.4 | Heat shock 70 kDa protein/Medicago truncatula/gi|357446059 | 244.33 | 236.9 | 561.8 ^a^ | 792 ^a^ |
| 69 | 144 | 14 | 90.0/5.34 | 100/5.2 | Heat-shock protein/Medicago truncatula/gi|357495325 | 95.6 | 526.83 ^a^ | 379.5 ^a^ | 466.1 ^a^ |
| 14 | 293 | 8 | 23.3/5.34 | 21/5.2 | Hsp23/Medicago sativa/gi|339792764 | 349 | 1547.6 ^a^ | 2984.73 ^a^ | 2753.9 ^a^ |
| 24 | 332 | 14 | 23.3/5.34 | 30/5.5 | Hsp23/Medicago sativa/gi|339792764 | 630.8 | 697.4 | 1015.56 ^a^ | 557.13 |
| 17 | 62 | 2 | 26.3/7.84 | 22/6.4 | Thaumatin-like protein/Capsicum annuum/gi|15419836 | 1101.46 | 1994.63 ^a^ | 1868.03 ^a^ | 1129.13 |
| 76 | 113 | 6 | 29.0/5.87 | 13/6.8 | Ubiquitin/Medicago truncatula/gi|357474747 | 558.4 | 555.6 | 1465.46 ^a^ | 614.9 |

Spot no. represents the number of proteins assigned.

PM shows the number of unique peptides matched.

The pI and molecular mass (Mr) values shown are the theoretical and observed values.

Protein function is assigned according to the NCBInr-protein sequence database.

^a^Indicates significant difference between control and treatments at the *P* < 0.05 level.
