# Supplementary material for: Proteomics Analysis of Alfalfa Response to Heat Stress
Source: PLoS One. 2013 Dec 6;8(12):e82725. doi: 10.1371/journal.pone.0082725 (PMC3855785; doi:10.1371/journal.pone.0082725)
Supplement: Table S2 — The homologs of unknown and hypothetical proteins identified by mass spectrometry between 24, 48 and 72 h heat stress (40°C) and normal temperature (25°C). (DOC) [file pone.0082725.s002.doc]

**Table 2** The homologs of unknown and hypothetical proteins identified by mass spectrometry between 24, 48 and 72 h heat stress (40°C) and normal temperature (25°C).

| **Spot no.** | **MOWSE score** | **PM** | **Theoretical(Mr/pI)** | **Observed (Mr/pI)** | **Protein name/Species/Acc.no.** | **Regulation** | | | | | | |
| --- | --- | --- | --- | --- | --- | --- | --- | --- | --- | --- | --- | --- |
| **02 Energy** | | | | | | | | | | | | |
| 77 | 92 | 2 | 14.4/8.93 | 14/5.5 | Cupin domain-containing protein/Arabidopsis thaliana /gi|15235021 | 720.6 | | 1370.86 ^a^ | | 1041.4 ^a^ | | 952.66 ^a^ |
| 40 | 288 | 7 | 42.9/8.46 | 44/5.5 | Glyceraldehyde-3-phosphate dehydrogenase A/Medicago truncatula /gi|357508529 | 458.16 | | 1387.66 ^a^ | | 871.7 ^a^ | | 2264.6 ^a^ |
| 93 | 242 | 10 | 27.5/6.77 | 35/5.9 | NAD-dependent dehydrogenase /Erythroxylum coca /gi|392056685 | 1295.6 | | 1471.03 | | 896.5 ^a^ | | 3442.16 ^a^ |
| **07 Transporters** | | | | | | | | | | | | |
| 47 | 406 | 16 | 57/4.95 | 79/5.2 | Protein disulfide isomerase-like protein precursor/Glycine max /gi|351722359 | 448.03 | | 846.7 ^a^ | | 1262.13 ^a^ | | 982.7 ^a^ |
| 53 | 110 | 7 | 29.4/7.77 | 32/8.0 | Porin/Prunus armeniaca/gi|5031279 | 869.53 | | 1139.96 ^a^ | | 2766 ^a^ | | 2135.76 ^a^ |
| **11 Disease/defence** | | | | | | | | | | | | |
| 90 | 434 | 14 | 27.1/9.02 | 28/5.6 | 20 kDa chaperonin/Glycine max/gi|356556406 | 1904.73 | 2377.27 ^a^ | | 3627.1 ^a^ | | 3959.4 ^a^ | |
| 25 | 104 | 4 | 23.4/5.81 | 24/5.3 | Dehydroascorbate reductase/Populus tomentosa/gi|405779437 | 385.2 | 269.7 ^a^ | | 1196.16 ^a^ | | 0 ^a^ | |
| 16 | 232 | 5 | 21.6/6.49 | 22/6.2 | Germin-like protein/Pisum sativum/gi|2739260 | 643.93 | 2483.53 ^a^ | | 2471.36 ^a^ | | 3428.66 ^a^ | |
| 31 | 188 | 18 | 38.8/8.18 | 35/6.0 | L-ascorbate peroxidase/Ricinus communis/gi|255545804 | 237.76 | 699.966 ^a^ | | 589.03 ^a^ | | 162.13 ^a^ | |
| 55 | 636 | 19 | 38.8/8.18 | 32/6.9 | L-ascorbate peroxidase/Ricinus communis/gi|255545804 | 913.1 | 1436.46 ^a^ | | 1650.83 ^a^ | | 1349.66 ^a^ | |
| 78 | 238 | 7 | 21.4/8.43 | 20/5.9 | Mitochondrial peroxiredoxin/Pisum sativum/gi|47775654 | 491.76 | 604.066 ^a^ | | 1151.16 ^a^ | | 784.9 ^a^ | |

For table layout, see the legends of Table 1.
